# Prevalence of HPV infection among 28,457 Chinese women in Yunnan Province, southwest China

**DOI:** 10.1038/srep21039

**Published:** 2016-02-12

**Authors:** Zheng Li, Feng Liu, Si Cheng, Lei Shi, Zhiling Yan, Jie Yang, Li Shi, Yufeng Yao, Yanbing Ma

**Affiliations:** 1 Department of Laboratory, Yan’an Hospital of Kunming, Kunming 650051, China; 2 Department of Laboratory, General Hospital of Yunnan Armed Police Force, Kunming 650111, China; 3 Institute of Medical Biology, Chinese Academy of Medical Sciences & Peking Union Medical College, Kunming 650118, China; 4 Department of Gynaecologic Oncology, The 3rd Affiliated Hospital of Kunming Medical University & Yunnan Tumour Hospital, Kunming 650118, China; 5 Wenzhou Medical University, Wenzhou 325035, China

## Abstract

Human papillomavirus (HPV) infection plays a key role in the development of cervical cancer. The aim of the current study was to investigate the HPV type distribution in Chinese women from Yunnan Province, southwest China. A total of 28,457 individuals ranging in age from 17–84 years were recruited from 13 clinical hospitals located in 10 different regions of Yunnan Province. Cervicovaginal swabs were collected from each participant, and HPV screening was performed using Luminex xMAP technology. Our results showed that the HPV prevalence was 12.9% in Yunnan Province. Overall, 10.6% of the individuals were positive for a single HPV type, and 2.3% were positive for multiple types. Among the individuals who tested positive for a single HPV type and multiple HPV types, the three most prevalent high-risk types were 52, 16, and 58. Age subgroup analysis showed two peaks for the frequencies of single and multiple HPV infections, one for the group of women under 25 years old, and the other for the group over 56 years old. Here, we present data regarding the prevalence and type distribution of HPV infection, which will aid in the estimation of the potential clinical benefit and cost-effectiveness of HPV screening and vaccination in China.

Human papillomavirus (HPV) has been identified as an aetiological factor for several anogenital diseases, particularly cervical cancer^1^,^2^. The outcome of HPV infection depends on its oncogenic type (low-risk or high-risk). The high-risk HPV types (HPV16, 18, 31, 33, 35, 39, 45, 52, 58 and 59) contribute to 96.6% of invasive cervical cancers diagnosed worldwide^2^. The low-risk types, such as HPV6, and 11, are associated with hyperplastic lesions.

As HPV plays an important role in the development of cervical cancer and other associated diseases, HPV screening is strongly advised because of its greater sensitivity and cost-effectiveness for detecting cervical cancer. For example, in China, HPV screening is more cost-efficient than cytology-based screening for cervical cancer^3^. HPV screening, especially for high-risk HPV, may reduce the risk of cervical cancer^3^,^4^.

Two HPV vaccines, a bivalent vaccine that targets HPV types 16 and 18 and a quadrivalent vaccine that targets types HPV6, 11, 16 and 18, are the most effective at reducing the risk of cervical cancer if they are administered prior to HPV exposure^5^,^6^,^7^. However, in addition to HPV16 and 18, the other high-risk types, including HPV31, 45, 52 and 58, account for approximately 10–20% of cervical cancers^2^. In addition, the limited cross-protection among HPV types^8^ and the heterogeneity in HPV type-specific distributions in different populations (for example, HPV52 and 58 are common types in high-grade squamous intraepithelial lesion (HSIL) and invasive cervical cancer (ICC) in China^9^,^10^,^11^ should be considered when predicting the effects of current prophylactic vaccines and developing new vaccines targeting an increased number of HPV types for more widespread application in more regions^12^.

Therefore, updated information on type-specific HPV prevalence and distribution in a given population is necessary for the development and evaluation of effective HPV vaccines and HPV screening tests. Yunnan Province is situated in southwest China and has a population of 36 million, and it is a relatively undeveloped region. Information regarding the prevalence and type distribution of HPV infection in Yunnan Province is incomplete, especially the prevalence of infection with multiple HPV types. We have previously investigated the prevalence of HPV in 5,376 female participants from Yunnan Province and have found a prevalence of 12.8%, revealing that the most common types are HPV52 and 16^13^. To confirm these results and to obtain additional information, in the current study, we examined a larger sample size (from 5,376 to 28,457) to assess the prevalence of HPV infection in Yunnan Province. Our investigation will provide data regarding the prevalence and type distribution of HPV infection in this region. In addition, our results will aid in the estimation of the potential clinical benefits and cost-effectiveness of HPV screening and vaccination in Yunnan Province.

## Materials and Methods

### Study population

The purpose of this investigation was to provide data on the prevalences of HPV genotypes, including age-specific prevalences and the prevalence of infections with multiple HPV genotypes. This investigation involved 13 clinical hospitals located in 10 different regions of Yunnan Province. A total of 28,539 Chinese women attending a gynaecological outpatient clinic and expressing a desire for access to cervical cancer screening were assessed between May 2011and July 2015. The inclusion criteria were as follows: women ① with a history of current or past sexual activity; ② who were not pregnant at the time of enrolment; ③ with no history of total uterus or cervical resection; ④ who were a permanent resident of the local area; and ⑤ who provided agreement to undergo an HPV test and participate in the present study. The investigation was approved by the Institutional Review Board of the Yan’an Hospital of Kunming. Written informed consent was obtained from each participant involved in the investigation. The methods of this investigation were in accordance with the approved guidelines and the principles expressed in the Declaration of Helsinki.

### Cervical specimen collection

Cervicovaginal swabs were collected from all participants by a gynaecologist according to the standard operation procedure for sampling at the recruitment sites. Before sampling, we ran a training course for the gynaecologists from different sampling sites. The swab was kept in 3 mL sample transport medium for the Tellgenplex™ HPV DNA Test (Tellgen Life Science, Shanghai, China). According to the Tellgenplex™ HPV DNA Test kit guidance, the samples could be stored in the sample transport medium for one week at room temperature, one month at 2–8 °C and six month at −20 °C. In order to get the accurate results, we require the sample to be transported at 4 °C within one week. All samples were shipped to the lab for HPV testing within 24 hours.

### HPV genotyping

The Tellgenplex™ HPV DNA Test uses a suspension bead array method to identify HPV types. The experimental protocol has beendescribed in a previous study^14^, and it involves PCR, bead-coated hybridization, flow cytometry, and the use of lasers and digital signal processing. HPV DNA-specific probes are coated on the surfaces of spectrally addressable polystyrene beads. As the beads are internally labelled with two spectrally distinct fluorophores, bead lots were assignable to class-specific HPV subtypes. Mixtures of different bead suspensions can be placed into the same well of a 96-well plate, allowing for multiplex analysis. The Tellgenplex™ HPV DNA Test can identify 26 HPV subtypes (HPV6, 11, 16, 18, 26, 31, 33, 35, 39, 40, 42, 44, 45, 51, 52, 53, 55, 56, 58, 59, 61, 66, 68, 73 82 and 83). Human β-globin was used as an internal control for each reaction.

### Data analysis

According to suggestions of the International Agency for Research on Cancer and the US Food and Drug Administration (FDA) (http://www.cdc.gov/mmwr/preview/mmwrhtml/rr6305a1.htm), 14 HPV types (HPV16, 18, 31, 33, 35, 39, 45, 51, 52, 56, 58, 59, 66, and 68) were classified as high-risk. The data were entered into an Excel spread sheet and then analysed using SPSS, and the overall and type-specific prevalences of HPV were calculated. A binomial 95% confidence interval (95% CI) was estimated for each calculation to get the prevalence of HPV. All genotypes from single and multiple infections were computed individually. These data were also stratified by age (≦25 years, 26–30 years, 31–35 years, 36–40 years, 41–45 years, 46–50 years, 51–55 years, and ≧56 years). Chi-squared tests were used to compare differences betweenage groups. P < 0.05 was considered statistically significant.

## Results

### Prevalence of HPV infection in Yunnan Province

A total of 28,457 individuals (average age of 36.69 ± 9.20) were included in the next analysis (out of 28,539 individuals). The remaining 82 individuals were excluded from further analysis because of a lack of results for human β-globin (internal control). Among these 28,457 individuals, positive HPV test results were obtained for 3,681 (average age of 36.17 ± 9.63), and negative results were observed for 24,776 (average age of 36.76 ± 9.14) (Table 1). Among the HPV-positive women, 3,017 were positive for a single HPV type (3,017/3,681 = 82.0% of HPV infections; 3,017/28,457 = 10.6% of all samples), and 664 were positive for multiple types (664/3,681 = 18.0% of HPV infections; 664/28,457 = 2.3% of all samples) (Table 1).

### The distribution and genotypes of HPV infections

Among the individuals who tested positive for a single HPV type, 89.1% (2,457/3,017 = 81.4%) were at high risk of HPV infection (Table 2). The four most prevalent high-risk types were HPV52, 16, 58 and 18, with frequencies of 2.1%, 1.7%, 1.0% and 0.6%, respectively (Table 2). In addition, the frequencies of the low-risk HPV types 6 and 11were 0.3% and 0.3%, respectively (Table 2). Among the 664 individuals infected with multiple HPV types, 533 had dual infections (533/664 = 80.3%), 97 had triple infections (97/664 = 14.6%), and 34 had four or more infections (34/664 = 5.1%). The four most prevalent high-risk HPV types were 52, 16, 58 and 39, with frequencies of 0.7%, 0.6%, 0.4% and 0.3%, respectively (Table 3).

### Age-specific prevalences of HPV infection

**Figure 1:**
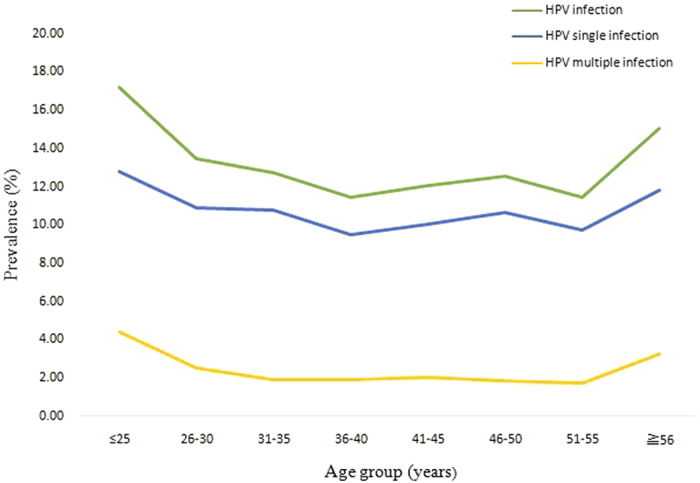
The prevalences of type-specific HPV infection in HPV-positive Chinese women (overall, single and multiple) in different age groups.

**Figure 2:**
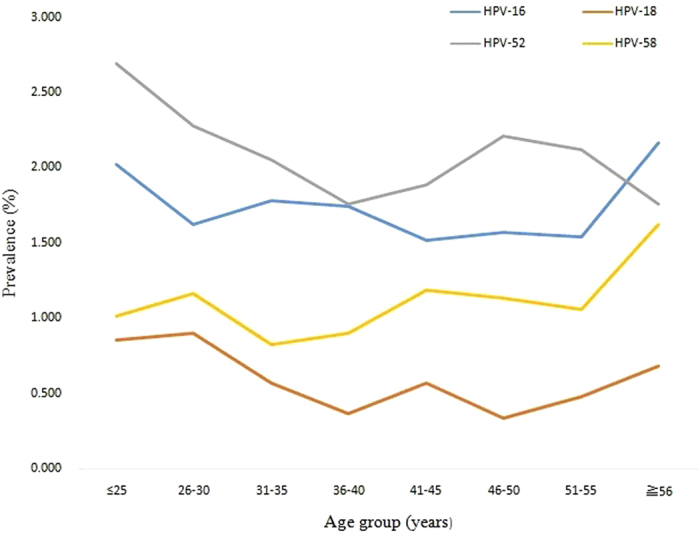
The percentages of single infections with four high-risk HPV subtypes (HPV16, 18, 52, and 58) in different age groups.

**Figure 3:**
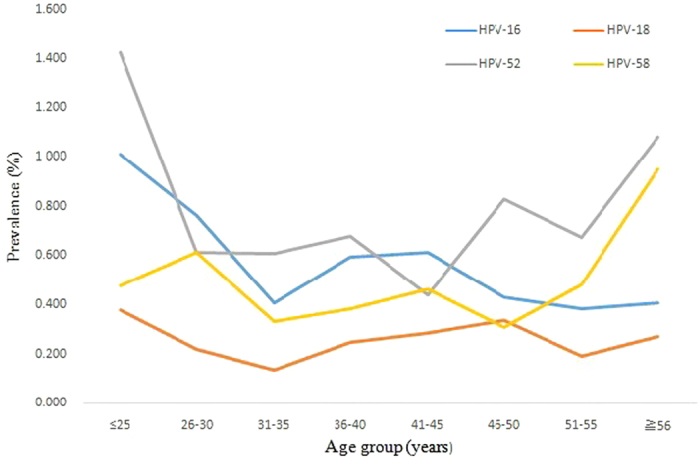
The percentages of multiple infections with four high-risk HPV subtypes (HPV16, 18, 52, and 58) in different age groups.

The prevalences of HPV infection were significantly different among the different age groups (χ2 =  72.69, P < 0.001), ranging from 11.43% for the women aged 36 to 40 years to 17.19% for those under 25 years of age (Fig. 1 and Table 4). Two peaks were observed for the presence of HPV infection, one for the group of women under 25 years old (543/3,159 = 17.2%) and the other for the group over 56 years old (111/738 = 15.0%) (Fig. 1). The prevalences of single and multiple HPV infections and high-risk single and multiple infections also exhibited two peaks, one for the group under 25 years old and the other for the group over 56 years old (Fig. 1). Figures 2 and 3 show that these age-related prevalences were also observed for the common high-risk HPV subtypes HPV16, 18, 52 and 58.

## Discussion

To date, two different prophylactic HPV vaccines have been developed to prevent cervical cancer, and they have both been proven to be effective for reducing the risk of cervical cancer. They are both licensed and available in most countries worldwide, except for Mainland China. Therefore, knowledge of the prevalences of specific HPV types will enable the development of optimal protective strategies in China.

In the current study, we examined the epidemiology of HPV and the prevalence of vaccine-type HPV infection in Yunnan Province, southwest China. Our results showed that the HPV prevalence was 12.9% in Yunnan Province. A total of 10.6% of the individuals studied tested positive for a single HPV type, and 2.3% tested positive for multiple types. The HPV prevalence in the current study is similar to previous (Supplementary Table 1)^10^,^15^,^16^,^17^,^18^. However, Wang *et al.*^19^ did a population- or employee-based cervical screening in 37 Chinese cities in 2015, and they reported that the total positive rate for hrHPV was 21.07% (18.42–31.94%), whose total rate for hrHPV was higher than the previous results^10^,^15^,^16^,^17^,^18^. The reason for this difference was hrHPV infection is becoming more serious and the infection rate is increasing in many regions, which indicated that those regions may soon reach the level for a heavy burden of infection^19^. Accordingly, the cervical cancer incidence has showed a continuous rise. For example, Wang *et al.*^20^ did an investigation about the incidence change and the epidemiological characteristics of cervical cancer in Beijing from 1993 to 2008. They observed the cervical cancer incidence has shown a continuous rise in Beijing since 1999. The other reason could be the advances in screening strategy, while the laboratory methods could also have partially contributed to the increased prevalence^19^. With regard to the age distribution, two peaks were observed for the presence of HPV infection in the current study, one for the group of women under 25 years old and the other for the group over 56 year old. These results are similar to those showed in other studies^10^,^17^,^19^,^21^,^22^. The sexual activity and immature immune protection for HPV of young women, and physiological and immunological disorders associated with hormone fluctuations during the menopausal transition of old women could explain why there is two peaks arise. Our result indicated that HPV detection is clinically valuable for women under 25 and over 56 years of age in cervical cancer screening programs.

Our results showed that the four high-risk HPV types, 52, 16, 58, and 18, were the most prevalent, similar to reports of HPV prevalence in other regions of China^9^,^17^,^22^,^23^,^24^ and in other Asian regions^2^. HPV16 and 18 are the most common types worldwide, and they account for approximately 70.9% of cervical cancers^2^. However, HPV52 and 58 are more prevalent in the Asian population, in both individuals with HPV infection and cervical cancer^25^,^26^,^27^. Particularly in China, HPV52 and 58 are more prevalent in the south and southwest among women with precancerous lesions and cervical cancer compared with the other regions^22^,^28^,^29^. The high prevalence of HPV52 and 58 in our study population confirm that future new vaccines against additional HPV types may offer a higher level of protection for women in China and in other Asian populations. Moreover, the frequencies of HPV16 and 18 in the patients with a single infection in this study were 16.1% and 5.5%, and those in the patients with multiple infections were 26.1% and 10.8%, respectively. The two currently licensed vaccines target HPV16 and 18; therefore, they would only cover 21.6% of single infection cases and 36.9% of multiple infection cases. Fortunately, Merck has recently submitted a Biologics License Application to the US FDA for an investigational nonavalent HPV vaccine, V503^30^. This nonavalent vaccine appears to be safe and effective in preventing persistent infection and precancerous lesions associated with HPV types 16/18/31/33/45/52/58 and genital warts related to types 6 and 11^30^. Based on our data, this new vaccine would cover 59.1% of single infection cases and 75.9% of multiple infections cases in Yunnan Province.

To date, several studies have shown that multiple HPV infections influence the duration of type-specific episodes and cervical cancer development^31^,^32^,^33^,^34^,^35^,^36^. For example, in 2001, Fifeet *et al.*^31^ reported that infection with multiple high-risk HPV types tends to increase the severity of cervical disease. Additionally, Trottier *et al.*^32^ have reported that HPV coinfections may influence viral oncogenic potential. Recently, Adela *et al.*^37^ has reported that coinfection with HPV68 and 16 increases the risks of HSIL and ICC compared with infection with either HPV16 or 68 alone. Thus, investigation of infections with multiple HPV types should improve our understanding of the prognosis of patients with persistent infection and the role of multiple infections in cervical cancer development. In the current study, the proportion of individuals with multiple infections among the HPV-positive women in Yunnan Province was 18.0%, which was lower than those reported in Shanghai (36.6%), Beijing (27.7%) and Shanxi (24.3%) and similar to those reported in Henan (19.8%) and Xinjiang (15.5%)^10^. In current study, two peaks were observed for the frequencies of high-risk HPV types in the patients with multiple infections, one for the group of women under 25 years old, and the other for the group over 56 years old. These results reflect the complex sexual relationships of younger individuals, which can account for the sexual transmission of multiple high-risk HPV genotypes. Moreover, our study also revealed that the frequency of high-risk HPV types in cases of multiple infections was high in the women over 56 years old. Several studies have reported an additional peak for the prevalence of multiple infections in individuals who are ≧50 or 60 years of age^10^,^17^,^21^,^22^. The results of our study and other studies suggest that the presence of this peak maybe attributed to the difficulty of older individuals in recovering from HPV infection or to viral persistence or reactivation of latent HPV caused by the physiologic and immunologic dysregulation that occurs during the menopausal transition^9^,^38^,^39^.

One limitation of the current study was the lack of behavioural information on the enrolled individuals. Some studies have reported that behaviours might increase the risks of cervical cancer and HPV infection, and the lack of such information in this study will make it difficult to perform future analyses for the roles of such exposure variables in the development of cervical cancer^40^,^41^. In addition, the current study focused only on the prevalence of HPV distribution in Yunnan province. The cytological data on the enrolled individualswere not collected in this study, thus, we did not have the data of cervical lesions classification, which may impact on base prevalence. Moreover, the sampling method in our current study is liquid-based cytology (LBC), which is still required for full validation for cervical sampling in comparison to other cervix directed methods. However, Howell-Jones *et al.*^42^ did a multi-site study of HPV type-specific prevalence in womenwith cervical cancer in England. They collected both LBC samplesand biopsy samples from a number of sites. They concluded that the strengths of their study including the collection of both LBC samples and biopsy samples from a number of sites geographically spread across England, and testing for the same HPV types^42^. In the future, a population-based study should be performed to investigate between the behavioural data and HPV infection, and the cervical lesions classification and HPV infection.

## Conclusions

In this study, we examined the epidemiology of HPV and the prevalence of vaccine-type HPV infection in Yunnan Province, southwest China. We have revealed the prevalences and distribution of the different HPV types, and this information will aid in the estimation of the potential clinical benefit and cost-effectiveness of HPV screening in Yunnan Province. Moreover, our results indicate that more high-risk HPV types (such as HPV52 and 58) should be considered in next-generation HPV prophylactic vaccines in the future.

## Additional Information

**How to cite this article**: Li, Z. *et al.* Prevalence of HPV infection among 28,457 Chinese women in Yunnan Province, southwest China. *Sci. Rep.* **6**, 21039; doi: 10.1038/srep21039 (2016).

## Supplementary Material

Supplementary Information

## Figures and Tables

**Table 1:** The Overall Prevalence of HPV in different age groups.

	Age group(year)								Total
	≦25	26–30	31–35	36–40	41–45	46–50	51–55	≧56	
HPV Positive (n)	543	613	649	689	549	408	119	111	3681
single infection	404	498	551	572	456	348	101	87	3017
multiple infection	139	115	98	117	93	60	18	24	664
HPV Negative (n)	2616	3954	4464	5340	4004	2851	920	627	24776
Total	3159	4567	5113	6029	4553	3259	1039	738	28457

Note: All genotypes from single and multiple infections were computed individually.

**Table 2:** The Overall Distribution of Single HPV Infections (N = 3017).

HPV type	Age group (year)								Infectious individuals (n)	Frequency for all samples (%)	95% CI for all samples (%)	Frequency for positive samples (%)	95% CI for positive samples (%)
	≦25	26–30	31–35	36–40	41–45	46–50	51–55	≧56					
6	23	17	10	10	5	8	4	4	81	0.28	0.22–0.34	2.20	1.73–2.67
11	11	15	15	16	8	7	0	2	74	0.26	0.20–0.32	2.01	1.56–2.46
16	64	74	91	105	69	51	16	16	486	1.71	1.56–1.86	13.20	12.11–14.30
18	27	41	29	22	26	11	5	5	166	0.58	0.49–0.67	4.51	3.84–5.18
26	0	3	0	3	1	0	0	0	7	0.02	0.00–0.04	0.19	0.05–0.33
31	7	13	25	22	25	9	3	2	106	0.37	0.30–0.44	2.88	2.34–3.42
33	16	11	20	23	20	12	2	3	107	0.38	0.31–0.45	2.91	2.36–3.45
35	4	5	10	5	6	3	4	0	37	0.13	0.09–0.17	1.01	0.68–1.33
39	18	22	32	29	16	14	3	6	140	0.49	0.41–0.57	3.80	3.19–4.42
40	3	4	4	4	4	1	1	1	22	0.08	0.05–0.11	0.60	0.35–0.85
42	2	2	4	8	7	3	2	0	28	0.10	0.06–0.14	0.76	0.48–1.04
44	5	9	8	10	2	9	1	1	45	0.16	0.11–0.21	1.22	0.87–1.58
45	3	7	6	5	6	3	0	1	31	0.11	0.07–0.15	0.84	0.55–1.14
51	10	18	18	11	16	16	1	0	90	0.32	0.25–0.39	2.44	1.95–2.94
52	85	104	105	106	86	72	22	13	593	2.08	1.91–2.25	16.11	14.92–17.30
53	13	17	18	15	15	15	3	2	98	0.34	0.27–0.41	2.66	2.14–3.18
55	5	6	8	9	11	8	2	2	51	0.18	0.13–0.23	1.39	1.01–1.76
56	15	19	24	28	22	18	9	5	140	0.49	0.41–0.57	3.80	3.19–4.42
58	32	53	42	54	54	37	11	12	295	1.04	0.88–1.12	8.01	7.14–8.89
59	11	14	9	17	18	7	2	5	83	0.29	0.23–0.35	2.25	1.78–2.73
61	13	7	8	18	11	18	4	0	79	0.28	0.22–0.34	2.15	1.68–2.61
66	8	9	9	12	6	5	2	3	54	0.19	0.15–0.25	1.47	1.08–1.86
68	17	20	35	27	13	13	3	1	129	0.45	0.37–0.53	3.50	2.91–4.10
73	0	0	0	0	0	0	0	0	0	0.00	0.00–0.00	0.00	0.00–0.00
82	11	7	20	12	8	7	1	3	69	0.24	0.18–0.30	1.87	1.44–2.31
83	1	1	1	1	1	1	0	0	6	0.02	0.04–0.04	0.16	0.03–0.29

Note: All genotypes from single and multiple infections were computed individually.

**Table 3:** The Overall Distribution of Multiple HPV Infections (N = 664).

HPV type	Age group (year)								Infectious individuals (n)	Frequency for all samples (%)	95% CI for all samples (%)	Frequency for positive samples (%)	95% CI for positive samples (%)
	≦25	26–30	31–35	36–40	41–45	46–50	51–55	≧56					
6	17	9	9	9	5	6	2	4	61	0.21	0.16–0.27	1.66	1.24–2.07
11	9	10	7	9	3	2	2	1	43	0.15	0.11–0.20	1.17	0.82–1.52
16	32	35	21	36	28	14	4	3	173	0.61	0.52–0.70	4.70	4.02–5.38
18	12	10	7	15	13	11	2	2	72	0.25	0.19–0.31	1.96	1.51–2.40
26	1	1	1	0	0	0	0	0	3	0.01	0.00–0.02	0.08	0.00–0.17
31	9	7	7	9	12	5	0	1	50	0.18	0.13–0.22	1.36	0.98–1.73
33	12	10	13	11	12	6	0	6	70	0.25	0.19–0.30	1.90	1.46–2.34
35	5	3	4	4	6	1	0	2	25	0.09	0.05–0.12	0.68	0.41–0.94
39	30	19	12	8	14	8	2	4	97	0.34	0.27–0.41	2.64	2.12–3.15
40	5	2	5	3	2	1	0	1	19	0.07	0.04–0.10	0.52	0.28–0.75
42	1	2	2	2	4	2	2	1	16	0.06	0.03–0.08	0.43	0.22–0.65
44	9	4	5	3	2	3	2	1	29	0.10	0.06–0.14	0.79	0.50–1.07
45	10	3	3	4	5	2	0	1	28	0.10	0.06–0.13	0.76	0.48–1.04
51	16	8	6	11	7	4	1	2	55	0.19	0.14–0.24	1.49	1.10–1.89
52	45	28	31	41	20	27	7	8	207	0.73	0.63–0.83	5.62	4.88–6.37
53	6	15	14	11	6	5	1	3	61	0.21	0.16–0.27	1.66	1.24–2.07
55	5	5	3	9	0	2	1	3	28	0.10	0.06–0.13	0.76	0.48–1.04
56	22	8	15	13	12	4	1	3	78	0.27	0.21–0.33	2.12	1.65–2.58
58	15	28	17	23	21	10	5	7	126	0.44	0.37–0.52	3.42	2.84–4.01
59	21	10	7	12	4	7	5	3	69	0.24	0.19–0.30	1.87	1.44–2.31
61	11	2	6	5	4	8	3	1	40	0.14	0.10–0.18	1.09	0.75–1.42
66	8	9	6	6	7	2	0	1	39	0.14	0.09–0.18	1.06	0.73–1.39
68	17	11	12	14	11	2	2	0	69	0.24	0.19–0.30	1.87	1.44–2.31
73	0	0	0	0	0	0	0	0	0	0.00	0.00–0.00	0.00	0.00–0.00
82	9	8	4	7	7	4	0	0	39	0.14	0.09–0.18	1.06	0.73–1.39
83	1	0	1	0	1	1	0	0	4	0.01	0.00–0.03	0.11	0.00–0.22

Note: All genotypes from single and multiple infections were computed individually.

**Table 4:** The statistically significant differences between the different age groups (*P* value).

	≦25	26–30	31–35	36–40	41–45	46–50	51–55	≧56
≦25		<0.001	<0.001	<0.001	<0.001	<0.001	<0.001	0.160
26–30			0.287	0.002	0.051	0.242	0.089	0.235
31–35				0.041	0.344	0.815	0.270	0.076
36–40					0.318	0.120	0.981	0.004
41–45						0.54	0.588	0.023
46–50							0.362	0.066
51–55								0.020
≧56								
