# Supplementary material for: Prevalence of HPV infection among 28,457 Chinese women in Yunnan Province, southwest China
Source: Sci Rep. 2016 Feb 12;6:21039. doi: 10.1038/srep21039 (PMC4751528; doi:10.1038/srep21039)
Supplement: Supplementary Information [file srep21039-s1.doc]

Prevalence of HPV infection among 28,457 Chinese women in Yunnan Province, southwest China

Zheng Li^1^*, Feng Liu^2^*, Si Cheng^1^, Lei Shi^3^, Zhiling Yan^4^, JieYang^5^, Li Shi^3^, Yufeng Yao^3^, Yanbing Ma^3^

1. Department of Laboratory, Yan’an Hospital of Kunming, Kunming 650051, China

2. Department of Laboratory, General Hospital of Yunnan Armed Police Force, Kunming 650111, China

3.Institute of Medical Biology, Chinese Academy of Medical Sciences & Peking Union Medical College, Kunming 650118, China

4. Department of Gynaecologic Oncology, The 3rd Affiliated Hospital of Kunming Medical University & Yunnan Tumour Hospital, Kunming 650118, China

5.Wenzhou Medical University, Wenzhou 325035,China.

Supplementary Table 1. HPV prevalence in women from population-based screening studies in China

| Author | Year | Location | Number(n) | Lab-assays | Overall HPV prevalence (%) | References |
| --- | --- | --- | --- | --- | --- | --- |
| Li et al. | This study | Yunnan | 28,457 | Tellgenplex™ HPV DNA Test | 12.9 |  |
| Li et al. | 2006 | Shenyang | 685 | MY09/11-based PCR assay | 16.8 | 15 |
| Zhao et al. | 2009 | Beijing | 5,552 | MY09/11 and reverse dot blot hybridisation | 6.7 | 16 |
| Ye et al. | 2010 | Zhejiang | 4987 | MY09/11-based PCR assay and HPV GenoArray Test kit | 13.3 | 18 |
| Wu et al. | 2013 | Beijing,Shanghai,Shanxi,Henan,Xinjiang | 4,215 | HC2-Linear Array | 14.3 | 10 |
| Jing et al. | 2014 | Guangdong | 78,355 | MassARRAY (Sequenom,Sandiego, CA) technique | 7.3 | 17 |
| Wang et al. | 2015 | Haikou | 692 | HC2 and Tellgenplex™HPV DNA Test | 31.9* | 19 |
| Wang et al. | 2015 | Chongqing | 700 | HC2 and Tellgenplex™HPV DNA Test | 27.3* | 19 |
| Wang et al. | 2015 | Jinan | 10,306 | HC2 and Tellgenplex™HPV DNA Test | 25.7* | 19 |
| Wang et al. | 2015 | Shenyang | 387 | HC2 and Tellgenplex™HPV DNA Test | 25.3* | 19 |
| Wang et al. | 2015 | Jilin | 1,423 | HC2 and Tellgenplex™HPV DNA Test | 25.3* | 19 |
| Wang et al. | 2015 | Tianjin | 3,220 | HC2 and Tellgenplex™HPV DNA Test | 25.1* | 19 |
| Wang et al. | 2015 | Shanghai | 522 | HC2 and Tellgenplex™HPV DNA Test | 22.6* | 19 |
| Wang et al. | 2015 | Nanning | 8,869 | HC2 and Tellgenplex™HPV DNA Test | 22.3* | 19 |
| Wang et al. | 2015 | Guiyang | 2,919 | HC2 and Tellgenplex™HPV DNA Test | 20.5* | 19 |
| Wang et al. | 2015 | Guangdong | 72,763 | HC2 and Tellgenplex™HPV DNA Test | 20.2* | 19 |
| Wang et al. | 2015 | Fuzhou | 2,213 | HC2 and Tellgenplex™HPV DNA Test | 19.9* | 19 |
| Wang et al. | 2015 | Chengdu | 1,886 | HC2 and Tellgenplex™HPV DNA Test | 19.9* | 19 |
| Wang et al. | 2015 | Hangzhou | 3,269 | HC2 and Tellgenplex™HPV DNA Test | 19.9* | 19 |
| Wang et al. | 2015 | Nanchang | 1,574 | HC2 and Tellgenplex™HPV DNA Test | 18.4* | 19 |

Note: * indicated the “ high-risk” HPV prevalence
